# Clinical, Virological, and Immunological Findings in Patients with Toscana Neuroinvasive Disease in Croatia: Report of Three Cases

**DOI:** 10.3390/tropicalmed5030144

**Published:** 2020-09-14

**Authors:** Tatjana Vilibic-Cavlek, Snjezana Zidovec-Lepej, Dragan Ledina, Samira Knezevic, Vladimir Savic, Irena Tabain, Ivo Ivic, Irena Slavuljica, Maja Bogdanic, Ivana Grgic, Lana Gorenec, Vladimir Stevanovic, Ljubo Barbic

**Affiliations:** 1 Department of Virology, Croatian Institute of Public Health, 10000 Zagreb, Croatia; irena.tabain@hzjz.hr (I.T.); maja.bogdanic11@gmail.com (M.B.); 2 School of Medicine, University of Zagreb, 10000 Zagreb, Croatia; 3 Department of Immunological and Molecular Diagnostics, University Hospital for Infectious Diseases “Dr Fran Mihaljevic”, 10000 Zagreb, Croatia; szidovec@gmail.com (S.Z.-L.); ivanahobby@gmail.com (I.G.); lgorenec@gmail.com (L.G.); 4 Clinic for Infectious Diseases, University Hospital Center Split, 21000 Split, Croatia; dledina@kbsplit.hr (D.L.); iivic@kbsplit.hr (I.I.); 5 School of Medicine, University of Split, 21000 Split, Croatia; 6 Infectious Disease Clinic, Clinical Hospital Center Rijeka, 51000 Rijeka, Croatia; samira.knezevic.rijeka@gmail.com (S.K.); irena.slavuljica@uniri.hr (I.S.); 7 Laboratory for Virology and Serology, Croatian Veterinary Institute, 10000 Zagreb, Croatia; v_savic@veinst.hr; 8 Faculty of Medicine, University of Rijeka, 51000 Rijeka, Croatia; 9 Department of Microbiology and Infectious Diseases with Clinic, Faculty of Veterinary Medicine University of Zagreb, 10000 Zagreb, Croatia; vladostevanovic@gmail.com (V.S.); ljbarbic@gmail.com (L.B.)

**Keywords:** Toscana virus, neuroinvasive disease, clinical presentation, virology, immunology, Croatia

## Abstract

Toscana virus (TOSV) is an arthropod-borne virus, transmitted to humans by phlebotomine sandflies. Although the majority of infections are asymptomatic, neuroinvasive disease may occur. We report three cases of neuroinvasive TOSV infection detected in Croatia. Two patients aged 21 and 54 years presented with meningitis, while a 22-year old patient presented with meningoencephalitis and right-sided brachial plexitis. Cerebrospinal fluid (CSF), serum, and urine samples were collected and tested for neuroinvasive arboviruses: tick-borne encephalitis, West Nile, Usutu, TOSV, Tahyna, and Bhanja virus. In addition, CSF and serum samples were tested for the anti-viral cytokine response. High titers of TOSV IgM (1000–3200) and IgG (3200−10,000) antibodies in serum samples confirmed TOSV infection. Antibodies to other phleboviruses (sandfly fever Sicilian/Naples/Cyprus virus) were negative. CSF samples showed high concentrations of interleukin 6 (IL-6; range 162.32−2683.90 pg/mL), interferon gamma (IFN-γ; range 110.12−1568.07 pg/mL), and IL-10 (range 28.08−858.91 pg/mL), while significantly lower cytokine production was observed in serum. Two patients recovered fully. The patient with a brachial plexitis improved significantly at discharge. The presented cases highlight the need of increasing awareness of a TOSV as a possible cause of aseptic meningitis/meningoencephalitis during summer months. Association of TOSV and brachial plexitis with long-term sequelae detected in one patient indicates the possibility of more severe disease, even in young patients.

## 1. Background

Toscana virus (TOSV) is an arthropod-borne virus, transmitted to humans by phlebotomine sandflies (*Phlebotomus* spp.). The virus can be transmitted transovarially in vectors, but its animal reservoir has not been identified yet. Three genetic lineages of TOSV, A, B, and C, have been identified so far. Although the seroprevalence studies indicate that the TOSV is endemic in the Mediterranean countries, the virus remains neglected since clinical cases of TOSV infection are rarely reported [1]. Infection rates are highest in summer months when the sandflies are most active. The majority of human TOSV infections are asymptomatic or presented as a non-specific febrile disease. However, neuroinvasive disease (meningitis, meningoencephalitis, encephalitis) may also occur [2]. Although self-resolving in most cases, TOSV infection of the central nervous system (CNS) may be severe in some patients [3,4]. Some rare or atypical clinical presentations caused by TOSV such as afebrile meningoencephalitis with transient central facial paralysis, aphasia, and paresis are also reported [5,6]. Since there are no clear clinical grounds to differentiate TOSV infections from other viral neuroinvasive infections, laboratory confirmation is required [7]. Diagnosis of TOSV can be confirmed by detection of TOSV RNA and/or detection of specific antibodies [8].

In Croatia, there is only one published report on clinical cases of TOSV infection. In 2007−2008, five cases of TOSV meningitis were confirmed at the Croatian littoral [9]. However, high seroprevalence rates were detected in 2012 among residents of Croatian islands (53.9%) and coastal area (33.6%), respectively. In addition, seropositive persons were also detected in the Croatian mainland (6.1%) indicating that TOSV is widespread in Croatia [10]. Phylogenetic analyses confirmed the co-circulation of two genetic lineages (B and C) in the coastal Croatian regions [9,11]. Anti-viral cytokine response was not measured.

We analyzed clinical, virological, and immunological findings in three cases of TOSV neuroinvasive infection detected during the two consecutive transmission seasons (2018−2019).

## 2. Case Reports

Patients’ demographic, epidemiological, and clinical data are presented in Table 1.

Case 1: In late August 2018, a 21-year-old male patient, inhabitant of the Croatian littoral was admitted to the Infectious Disease Clinic, University Hospital Center Split with a two-day history of fever (up to 38 °C), headache, nausea, vomiting, photophobia, and weakness. Physical examination was normal. Routine laboratory tests were normal. Cerebrospinal fluid (CSF) analysis revealed a WBC count of 175 cells/mm^3^ (76% lymphocytes), a protein level of 0.447 g/L (reference range 0.17–0.37 g/L), and a glucose level of 3.78 mmol/L (reference range 2.5−3.3 mmol/L). Brain computed tomography (CT) was normal. The patient recovered fully within few days.

Case 2: In late August 2019, a 22-year old female patient was admitted to the Infectious Disease Clinic, University Hospital Center Split with an eight-day history of fever (up to 38 °C), headache, nausea vomiting, photophobia, dizziness, and weakness. On the 6th day after disease onset, a maculopapular rash developed with arthralgia (wrists and ankles). Physical examination showed neck stiffness and right upper arm neuralgic pain that limited arm mobility and was diagnosed as brachial plexitis. Routine laboratory tests were normal. CSF analysis revealed a WBC count of 102 cells/mm^3^ (76% lymphocytes), a protein level of 0.993 g/L, and a glucose level of 3.0 mmol/L. Brain magnetic resonance imaging (MRI) showed two hyperintensities in the left frontal lobe (unidentified bright objects). Right brachial plexus MRI finding was normal and there were no signs of denervation on electroneurography. After three-week hospitalization symptoms improved and the patient was discharged with moderate pain and improved upper arm mobility.

Case 3: In mid-October 2019, a 54-year-old male patient was admitted to the Infectious Disease Clinic, Clinical Hospital Center Rijeka with a one-day history of severe headache, vomiting, and weakness. A week before, during his stay on Solta Island, he noticed redness and swelling on his face after a mosquito bite. Physical examination showed neck stiffness. Routine laboratory tests were normal. CSF analysis revealed a WBC count of 123 cells/mm^3^ (86% lymphocytes), a protein level of 1.065 g/L, and a glucose level of 4.05 mmol/L. Brain CT and CT angiography were normal. The patient recovered fully within few days.

### 2.1. Virology Results

Since the CSF findings were suggestive of aseptic meningitis, arboviral etiology was suspected. CSF, serum, and urine samples were collected and tested for the presence of neuroinvasive arboviruses: tick-borne encephalitis virus (TBEV), West Nile (WNV), Usutu (USUV), TOSV, Tahyna (TAHV), and Bhanja virus (BHAV). To exclude cross-reactivity with other phleboviruses, samples were also tested for sandfly fever Naples (SFNV), Sicilian (SFSV), and Cyprus (SFVC) virus. Serological tests were performed using commercial enzyme-linked immunosorbent assays (ELISA; TBEV, WNV, USUV, Euroimmun, Lübeck, Germany) or indirect immunofluorescence assays (IFA; sandfly fever mosaic, Euroimmun, Lübeck, Germany). In addition, CSF and serum samples were tested for the presence of viral RNA: TBEV [12], WNV [13], USUV [14], TOSV [15], TAHV [16], and BHAV [17].

**Figure 1:**
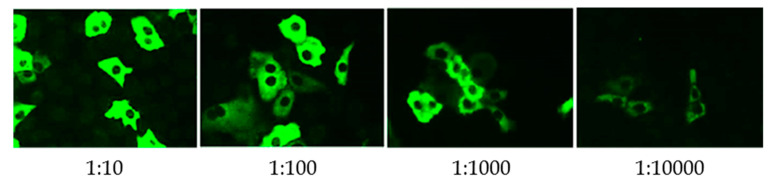
Toscana virus IgG antibodies detected using indirect immunofluorescence assay in a patient (case 3) with neuroinvasive infection.

Virology results are presented in Table 2. High titers of both IgM and IgG to TOSV in serum samples indicated acute TOSV infection (Figure 1). TOSV RNA was not detected in CSF, serum, and urine samples by use of highly sensitive and specific real-time reverse transcriptase polymerase chain reaction (RT-qPCR) [15]. Antibodies to other phleboviruses were negative.

### 2.2. Anti-Viral Cytokine Response

To analyze the anti-viral cytokine response, a commercial multiplex assay was used for simultaneous detection of 13 cytokines in the CSF and serum samples: IL-5,IL-13, IL-2, IL-6, IL-9, IL-10, IFN-γ, TNF-α, IL-17A, IL-17F, IL-4, IL-21, and IL-22 (LEGENDplex™ Human Anti-Virus Response Panel, BioLegend, San Diego, CA, USA). In the CSF samples of all three cases, high concentrations of IL-6 (range 162.32−2683.90 pg/mL), IFN-γ (range 110.12−1568.07 pg/mL), and IL-10 (range 28.08−858.91 pg/mL) were found. Two samples (cases 2 and 3) showed elevated levels of IL-22 (28.49 and 45.24 pg/mL, respectively) as well, while one sample (case 3) showed elevated level of IL-13 (40.05 pg/mL). In serum samples, concentrations of cytokines were significantly lower than in CSF (Table 3).

## 3. Discussion

In Croatia, TBEV and WNV are the most commonly detected neuroinvasive arboviruses. Sporadic cases of USUV neuroinvasive disease were also reported [18,19,20]. So far, only few cases of TOSV infection are diagnosed and it is not a routine consideration in the differential diagnosis of viral meningoencephalitis. Since the TOSV seroprevalence rates are high in the inhabitants of the Croatian littoral, the true prevalence of the disease is probably underestimated.

Cases presented in this report developed symptoms of neuroinvasive disease in late August and mid-October. Taking into account the area of residence with documented high seropositivity to TOSV [10] and arbovirus transmission season, TOSV infection was suggested. In all patients, infection was confirmed by detection of high titers of both TOSV IgM and IgG antibodies, while qRT-PCR was negative. The results of a recently published study showed that specific antibody response develops rapidly in neuroinvasive TOSV infection. TOSV IgM and IgG antibodies were present at the onset of symptoms in 100% of patients [21], which could explain the negative RT-qPCR result in serum and CSF samples of the patients presented in this report.

Data on cytokine levels during TOSV infection are scarce [22,23]. In this study, a very high levels of IL-6, IFN-γ, and IL-10 were found in all three CSF samples, while in serum samples levels of cytokines were significantly lower. Moderate increase of IL-6, IFN-γ, and IL-10 was found in two and one serum samples, respectively. In one recently published study, increased levels of IFN-γ and IL-22 were found in serum samples of patients with TOSV infection while CSF samples were not available for testing [22]. None of the serum samples in the present study showed elevated levels of IL-22, however, elevated levels were detected in two CSF samples. In the other study, no difference was observed in the plasma levels of IL-6, IL-10, TNF-α, IFN-α, and IFN-γ between TOSV-infected patients and controls, however, levels of cytokines in the CSF was significantly higher in TOSV-infected patients. No difference was found in the CSF cytokine production in patients with meningitis and meningoencephalitis [23]. Similarly, our results showed no association of inflammatory markers in the CSF and disease severity. A high intrathecal production of IL-6, IFN-γ, and IL-10 suggests the activation of a selective antiviral and anti-inflammatory response in the CNS of TOSV-infected patients which is in correlation with observations in some other neuroinvasive arboviral infections (flaviviruses) where a strong inflammatory immune response in the CNS was found [23,24,25].

Two patients in this report presented with meningitis, while one patient presented with meningoencephalitis and brachial plexitis. Although the etiology of brachial plexitis is unknown, a viral etiology (association with recent viral infection or vaccination) was proposed. Viral causes reported so far include cytomegalovirus, coxsackieviruses, varicella-zoster virus, Epstein–Barr virus, and parvovirus B19. Recent viral infection has been associated to precede the development of the disease in 25−55% of patients [26,27].

Although clinical outcome of TOSV neuroinvasive disease is usually favorable with no long-term sequelae, compared to other arboviral infections such as TBEV and WNV, severe and fatal encephalitis was reported in elderly [28]. Despite a full recovery within a few weeks in most cases of TOSV infection [29], as in the two patients reported in this study, the presented cases highlight the need of increasing awareness of TOSV as a possible cause of aseptic meningitis/meningoencephalitis. The long-term sequelae (brachial plexitis) detected in one patient indicates the possibility of more severe disease, even in young patients.

Due to the similar clinical presentation and seasonal distribution, TOSV infection can be misdiagnosed with some other neuroinvasive viral infections such as TBEV, WNV, USUV [18,19,20], and enteroviruses [30]. In countries where several arboviruses co-circulate, TOSV should be included in the differential diagnosis of aseptic meningitis/meningoencephalitis, especially during the summer months.

## 4. Conclusions

Clinicians should consider TOSV in differential diagnosis of neuroinvasive disease during the arbovirus transmission season. Since Croatia is a touristic country, TOSV should also be considered as one of the viral pathogens causing aseptic meningitis in travelers returning from this area.

## Figures and Tables

**Table 1:** Epidemiological and Clinical Characteristics of Patients with Toscana Neuroinvasive Infection.

Characteristic	Case 1	Case 2	Case 3
Age	21 years	22 years	54 years
Gender	Male	Female	Male
Area of residence	Croatian littoral (Middle Dalmatia)	Croatian mainland (worked at the Croatian littoral; Middle Dalmatia from July 2019)	Croatian littoral (stayed on holiday in Middle Dalmatia)
Time of disease onset	Late August	Late August	Mid October
Clinical presentation	Meningitis	Meningoencephalitis	Meningitis
Clinical symptoms	Fever (up to 38 °C), headache, nausea, vomiting, photophobia, weakness	Fever (up to 38 °C), headache, nausea, vomiting, photophobia, dizziness, weakness, arthralgia, maculopapular rash, right-sided brachial plexitis	Severe headache, vomiting, weakness
Duration of symptoms	5 days	20 days	5 days
Outcome	Recovered	Improved	Recovered

**Table 2:** Virology Results in Patients with Toscana Neuroinvasive Infection.

Virus	CASE 1			CASE 2			CASE 3		
	ELISA IgM ^a^/IgG ^b^	IFA IgM/IgG ^c^	RT-qPCR	ELISA IgM ^a^/IgG ^b^	IFA IgM/IgG ^c^	RT-qPCR	ELISA IgM ^a^/IgG ^b^	IFA IgM/IgG ^c^	RT-qPCR
TBEV	NegNeg	NT	Neg	Neg/Neg	NT	Neg	Neg/Neg	NT	Neg
WNV	Neg/Neg	NT	Neg	Neg/Neg	NT	Neg	Neg/Neg	NT	Neg
USUV	NT/Neg	NT	Neg	NT/Neg	NT	Neg	NT/Neg	NT	Neg
SFSV	NT	Neg/Neg	NT	NT	Neg/Neg	NT	NT	Neg/Neg	NT
SFNV	NT	Neg/Neg	NT	NT	Neg/Neg	NT	NT	Neg/Neg	NT
SFCV	NT	Neg/Neg	NT	NT	Neg/Neg	NT	NT	Neg/Neg	NT
TOSV	NT	1000/3200	Neg	NT	3200/3200	Neg	NT	1000/10,000	Neg
TAHV	NT	NT	Neg	NT	NT	Neg	NT	NT	Neg
BHAV	NT	NT	Neg	NT	NT	Neg	NT	NT	Neg

TBEV = tick-borne encephalitis virus; WNV = West Nile virus; USUV = Usutu virus; SFSV = sandfly fever Sicilian virus; SFNV = sandfly fever Naples virus; SFCV = sandfly fever Cyprus virus; TOSV = Toscana virus; TAHV = Tahyna virus; BHAV = Bhanja virus; NT = not tested; ^a^ ratio < 0.8 negative, 0.8−1.1 borderline, ≥ 1.1 positive; ^b^ RU/mL < 16 negative, 16−22 borderline, ≥ 22 positive; ^c^ titer < 100 negative.

**Table 3:** Anti-Viral Cytokine Levels in Patients with Toscana Virus Neuroinvasive Infection.

Cytokine (pg/mL)	Case 1		Case 2		Case 3		Detection Limits (Minimum Detectable Concentrations + 2xSD, pg/mL)
	Serum	CSF	Serum	CSF	Serum	CSF	
IL-5	Neg	Neg	5.35	Neg	Neg	3.20	1.2 + 1.3
IL-13	Neg	Neg	18.68	Neg	71.25	40.05	1.4 + 0.7
IL-2	5.92	Neg	Neg	Neg	Neg	35.01	1.4 + 0.4
IL-6	Neg	162.32	8.35	517.32	9.29	2683.90	1.0 + 0.8
IL-9	5.51	5.01	4.80	2.13	Neg	7.62	1.7 + 1.4
IL-10	Neg	199.99	Neg	28.08	Neg	858.91	0.7 + 0.4
IFN-γ	Neg	110.12	28.23	206.92	Neg	1568.07	1.1 + 0.7
TNF-α	13.75	Neg	Neg	Neg	Neg	Neg	0.7 + 0.5
IL-17A	Neg	Neg	32.94	Neg	Neg	Neg	1.9 + 0.6
IL-17F	15.21	Neg	Neg	Neg	Neg	Neg	0.8 + 0.7
IL-4	18.43	Neg	5.97	Neg	Neg	Neg	1.0 + 0.8
IL-21	78.13	Neg	10.98	Neg	Neg	Neg	6.0 + 3.0
IL-22	Neg	Neg	Neg	28.49	Neg	45.24	1.5 + 0.5
